# Forest fire smoke as a threat to the health of river dolphins

**DOI:** 10.1111/cobi.70098

**Published:** 2025-06-24

**Authors:** Enzo Aliaga‐Rossel, David Edinger, Miriam Marmontel, Luis Guizada Duran, Andreas Fahlman

**Affiliations:** ^1^ Instituto de Ecología Universidad Mayor de San Andrés La Paz Bolivia; ^2^ Program of Conservation and Research of River dolphins in Bolivia La Paz Bolivia; ^3^ Instituto de Desenvolvimento Sustentável Mamirauá Tefé Brazil; ^4^ Programa de Pós‐Graduação em Biodiversidade e Conservação da Natureza Universidade Federal Juiz de Fora Juiz de Fora Brazil; ^5^ IFM Linköping University Linköping Sweden; ^6^ Fundación Oceanogràfic de la Comunitat Valenciana Valencia Spain; ^7^ Global Diving Research SL Sanlucar de Barrameda Spain; ^8^ Woods Hole Oceanographic Institution, Woods Hole MA USA

**Keywords:** Amazon basin, aquatic mammal, cetacean, conservation, forest fires, Inia, respiratory disease

South American river dolphins (genus *Inia* d'Orbigny, 1834) (also known as botos or bufeos) are widely distributed across the rivers of the Amazon and Orinoco basins. However, their conservation status is compromised by declining populations, which is pushing them toward extinction (da Silva et al., 2018). Like other cetaceans, botos are particularly prone to respiratory problems, including flu and pulmonary infections (Best & da Silva, 1993; Bonar et al., 2007; Rodrigues et al., 2018). It is not uncommon to find individuals naturally affected by these ailments.

The Amazon basin is one of the most biodiverse biomes on the planet (Aliaga‐Rossel et al., 2023; Hoorn et al., 2010). However, the decline in biodiversity in the basin is reaching a tipping point (Feng et al., 2021). A recent and escalating threat is the large‐scale fires in the Amazon, which increased significantly in frequency and extent from 2019 to 2024 (Armenteras et al. 2021). These fires are intentionally set to clear land and are primarily driven by agroindustrial expansion, weak or nonexistent enforcement of agricultural regulations, rampant land trafficking, and political motives (Armenteras & de la Barrera, 2023). This practice further degrades the already fragile habitat of the boto, exacerbating the pressures on these species (Feng et al., 2021; Genov, 2021).

The timing of the forest fires in the Amazon region coincides with the dry season, when rivers experience significant reductions in flow, particularly in the headwaters of the basins. In addition, severe droughts occurred in the Amazon region in 2023 and 2024, resulting in conditions that exacerbated and extended forest fires. For example, in Bolivia alone, it is estimated that at least 12 million ha were lost to fire in 2024 (Fundación Tierra, 2024).

Forest fires produce thick, dense smoke made up of harmful gases, such as carbon monoxide, and aerosolized particles and other toxic chemical compounds (Urbanski et al., 2008). This smoke has long been associated with increased risks of respiratory and cardiovascular illnesses in humans (Padamsey et al., 2024; Zhang et al., 2025; cdc.gov). For example, in 2024, the severity of the fires in Bolivia and the resulting dangerously poor air quality led to unprecedented measures, including the closure of airports and schools for over 6 weeks in several cities. The air quality index for September to October 2024 was reported as a hazard for the region (https://www.iqair.com) and highlighted the smoke's devastating health impacts, such as the worsening of respiratory conditions, aggravation of chronic cardiorespiratory diseases, and even the death of an infant (Red Uno, 2024; Soruco‐Ruiz, 2024).

Frequent forest fire smoke not only affects the air quality for human populations but also imposes significant risks to wildlife. However, aquatic mammals, such as river dolphins, are particularly vulnerable due to their limited ability to migrate (Da Silva et al., 2018). In addition, their respiratory anatomy, adapted for breath‐hold diving, makes them particularly susceptible to inhalation of volatile compounds or aerosolized particles in smoke (Piscitelli‐Doshkov et al., 2024). For example, inhalation of volatile petroleum compounds following the *Deepwater Horizon* oil spill resulted in significant moderate to severe respiratory sequelae in bottlenose dolphins *Tursiops truncatus* (Montagu, 1821), such as alveolar interstitial syndrome, pleural effusion, and consolidation, that persisted for at least 8 years following the accident (Smith et al., 2022). These respiratory issues resulted in an annual mortality that was 300% higher than in areas without exposure to oil (Genov, 2021; Smith et al., 2022). Prolonged exposure to smoke likely affects dolphin respiratory systems, increasing the probability of respiratory diseases, such as pneumonia (Fitzgerald & Flood, 2006). Because dolphins use their lungs to replenish oxygen between dives, respiratory disease is likely to reduce their diving capacity and ability to obtain food (Shero et al., 2024; Wells & Fahlman, 2024). The effect of smoke inhalation has been reported in bottlenose dolphins. For example, there is a 3.5 times higher likelihood of death following bacterial pneumonia in years with wildfire (Venn‐Watson et al., 2013). Furthermore, smoke will secondarily affect botos’ ability to nurse and care for their newborns and calves, which are born during fire season (McGuire & Aliaga‐Rossel, 2007). The calves undergo a process to master controlled breathing, and while their lungs are developing, they breathe more frequently, making them particularly vulnerable to poor air quality.

During the dry season, river dolphins are often confined to lagoons and deep meanders, limiting their available habitats and restricting their ability to move to safer locations (Aliaga‐Rossel & Guizada, 2020). Casual observations of river dolphins sneezing or coughing in areas heavily affected by fire suggest respiratory distress caused by smoke. Diminished respiratory function, combined with compromised air quality, likely weakens their immune systems, reducing fitness, long‐term survival, and quality of life in the Amazon. In one study in San Diego (USA), Venn‐Watson et al. (2013) found that older dolphins show a higher prevalence of bacterial or fungal pneumonia at death following fires. Older animals may therefore be especially vulnerable because smoke inhalation can exacerbate underlying health issues.

Because fires are increasing in intensity and frequency in the Amazon and worldwide, it is imperative for authorities to act swiftly to prevent severe declines in populations of already threatened species (Campbell et al., 2022; Guizada et al., 2024). Future research is essential to systematically assess the impacts of forest fires and smoke on river dolphins, particularly through histopathological analyses of necropsy samples, to determine whether fire‐related pollutants contribute to illness and mortality. In addition, noninvasive lung function testing works well to evaluate respiratory health and could be a powerful diagnostic tool for early detection and intervention of affected wild dolphins (Fahlman et al., 2025).

The Amazon basin, one of the world's most biodiverse ecosystems, plays a critical role in global ecological stability. It acts as a carbon sink, plays a key role in water cycles, regulates climate, and is home to endemic species; therefore, it is essential for maintaining global biodiversity and ecosystem functioning.

## References

[cobi70098-bib-0001] Aliaga‐Rossel, E. , & Guizada, L. A. (2020). Bolivian river dolphin site preference in the middle‐section of Mamoré River, upper Madeira river basin, Bolivia. Therya, 11(3), 459–465.

[cobi70098-bib-0002] Aliaga‐Rossel, E. , Martins, M. B. , Barrera, S. , Benítez, Á. , Cano, C. A. , dos Santos, T. C. M. , Díaz, V. , Domínguez, J. H. , Domínguez‐López, M. E. , Lopes, G. C. S. , Escobar, M. D. , Souza, V. F. , Freitas, V. , Gidsicki, D. , Guerra, D. , Hidalgo, M. , Lopes, G. P. , Trentin, I. C. , Lozano Báez, S. E. , … Paredes, G. (2023). Capítulo 2: Situación, tendencias y dinámica de la diversidad biológica y las contribuciones de la naturaleza para las personas. In M. E. Corvalán (Ed.), Evaluación Rápida de la Diversidad Biológica y Servicios Ecosistémicos en la Región Amazónica (pp. 15–167). OTCA, Proyecto OTCA/BIOMAZ, GIZ‐Brasil, Ministerio Federal Alemán de Cooperación Económica y Desarrollo (BMZ), Instituto Humboldt (Colombia).

[cobi70098-bib-0003] Armenteras, D. , Dávalos, L. M. , Barreto, J. S. , Miranda, A. , Hernández‐Moreno, A. , Zamorano‐Elgueta, C. , González‐Delgado, T. M. , Meza‐Elizalde, M. C. , & Retana, J. (2021). Fire‐induced loss of the world's most biodiverse forests in Latin America. Science Advances, 7, Article eabd3357. 34389532 10.1126/sciadv.abd3357 PMC8363147

[cobi70098-bib-0004] Armenteras, D. , & de la Barrera, F. (2023). Landscape management is urgently needed to address the rise of megafires in South America. Communications Earth & Environment, 4, Article 305.

[cobi70098-bib-0005] Best, R. C. , & da Silva, V. M. F. (1993). Inia geoffrensis. Mammalian Species, 426, 1–8.

[cobi70098-bib-0006] Bonar, C. J. , Boede, E. O. , Hartmann, M. G. , Lowenstein‐Whaley, J. , Mujica‐Jorquera, E. , Parish, S. V. , Parish, J. V. , Garner, M. M. , & Stadler, C. K. (2007). A retrospective study of pathologic findings in the Amazon and Orinoco river dolphin (*Inia geoffrensis*) in captivity. Journal of Zoo and Wildlife Medicine, 38(2), 177–191. 17679501 10.1638/1042-7260(2007)038[0177:ARSOPF]2.0.CO;2

[cobi70098-bib-0007] Campbell, E. , Alfaro‐Shigueto, J. , Aliaga‐Rossel, E. , Beasley, I. , Briceño, Y. , Caballero, S. , da Silva, V. M. F. , Gilleman, C. , Gravena, W. , Hines, E. , Shahnawaz Khan, M. , Khan, U. , Kreb, D. , Mangel, J. C. , Marmontel, M. , Mei, Z. , Mintzer, V. J. , Mosquera‐Guerra, F. , Oliveira‐daCosta, M. O. , … Godley, B. J. (2022). Challenges and priorities for river cetacean conservation. Endangered Species Research, 49, 13–42.

[cobi70098-bib-0008] da Silva, V. F. , Trujillo, F. , Martin, A. , Zerbini, A. N. , Crespo, E. , Aliaga‐ Rossel, E. , & Reeves, R. (2018). Inia geoffrensis. The IUCN Red List of Threatened Species 2018: e.T10831A50358152. International Union for Conservation of Nature.

[cobi70098-bib-0009] d'Orbigny, A. (1834). Notice sur un nouveau genre de cétacé, des rivières du centre de l'Amérique méridionale. Nouvelles annales du Muséum d'histoire naturelle, 3, 28–36.

[cobi70098-bib-0010] Fahlman, A. , Sterba‐Boatwright, B. , Cauture, F. , Sweeney, J. , & Stone, R. (2025). Spirometry as a diagnostic tool to assess respiratory health in beached bottlenose dolphins (*Tursiops* spp.). Diseases of Aquatic Organisms, 161, 113–124. 40110737 10.3354/dao03843

[cobi70098-bib-0011] Feng, X. , Merow, C. , Liu, Z. , Park, D. S. , Roehrdanz, P. R. , Maitner, B. , Newman, E. A. , Boyle, B. L. , Lien, A. , Burger, J. R. , Pires, M. M. , Brando, P. M. , Bush, M. B. , McMichael, C. N. H. , Neves, D. M. , Nikolopoulos, E. I. , Saleska, S. R. , Hannah, L. , Breshears, D. D. , … Enquist, B. J. (2021). How deregulation, drought and increasing fire impact Amazonian biodiversity. Nature, 597(7877), 516–521. 34471291 10.1038/s41586-021-03876-7

[cobi70098-bib-0012] Fitzgerald, K. T. , & Flood, A. A. (2006). Smoke inhalation. Clinical Techniques in Small Animal Practice, 21, 205–214. 17265906 10.1053/j.ctsap.2006.10.009

[cobi70098-bib-0013] Fundación Tierra . (2024). Reporte de incendios forestales en Bolivia. Informe técnico. https://www.ftierra.org/index.php/publicacion/documentos‐de‐trabajo/254‐reporte‐de‐incendios‐forestales‐en‐bolivia

[cobi70098-bib-0014] Genov, T. (2021). The impacts of chemical pollutants on cetaceans in Europe. In L. Nunny (Ed.), Under pressure: The need to protect whales and dolphins in European waters (pp. 110–118). OceanCare.

[cobi70098-bib-0015] Guizada Duran, L. A. , Aliaga‐Rossel, E. , Paschoalini Frias, M. P. , & Zerbini, A. N. (2024). Bolivian River Dolphin trends: A long‐term analysis in the Mamore basin. PLoS ONE, 19(10), Article e0308806. 39365787 10.1371/journal.pone.0308806 PMC11452032

[cobi70098-bib-0016] Hoorn, C. , Wesselingh, F. P. , ter Steege, H. , Bermudez, M. A. , Mora, A. , Sevink, J. , Sanmartín, I. , Sanchez‐Meseguer, A. , Anderson, C. L. , Figueiredo, J. P. , Jaramillo, C. , Riff, D. , Negri, F. R. , Hooghiemstra, J. , Lundberg, J. , Stadler, T. , Särkinen, T. , & Antonelli, A. (2010). Amazonia through time: Andean uplift, climate change, landscape evolution, and biodiversity. Science, 330, 927–931. 21071659 10.1126/science.1194585

[cobi70098-bib-0017] McGuire, T. , & Aliaga‐Rossel, E. (2007). Seasonality of reproduction in Amazon River dolphins (*Inia geoffrensis*) in three major river basins of South America. Biotropica, 39(1), 129–135.

[cobi70098-bib-0018] Montagu, G. (1821). Description of a species of Delphinus, which appears to be new. Memoirs of the Wernerian Natural History Society, 3, 75–82. https://www.biodiversitylibrary.org/page/45888588#page/103/mode/1up

[cobi70098-bib-0019] Padamsey, K. , Liebenberg, A. , Wallace, R. , & Oosthuizen, J. (2024). Characterizing the chemical composition of bushfire smoke and implications for firefighter exposure in Western Australia. Fire, 7(11), Article 388.

[cobi70098-bib-0020] Piscitelli‐Doshkov, M. , Kooyman, G. L. , & Fahlman, A. (2024). Respiratory physiology in the dolphin and other whales. In A. Fahlman & S. K. Hooker (Eds.), The physiology of dolphins (pp. 107–133). Academic press.

[cobi70098-bib-0021] Red Uno . (2024). Velan al niño que falleció por el humo de los incendios en la comunidad Río Blanco . https://www.reduno.com.bo/noticias/velan‐al‐nino‐que‐fallecio‐por‐el‐humo‐de‐los‐incendios‐en‐la‐comunidad‐rio‐blanco‐202492784734

[cobi70098-bib-0022] Rodrigues, T. C. S. , Díaz‐Delgado, J. , Catão‐Dias, J. L. , Carvalho, L. L. , & Marmontel, M. (2018). Retrospective pathological survey of pulmonary disease in free‐ranging Amazon river dolphin *Inia geoffrensis* and tucuxi *Sotalia fluviatilis* . Diseases of Aquatic Organisms, 131, 1–11. 30324910 10.3354/dao03280

[cobi70098-bib-0023] Shero, M. R. , Costa, D. P. , Burns, J. M. , & Goetz, K. (2024). Breath‐hold capacities and circadian dive rhythmicity shape optimal foraging strategies in a polar marine mammal, the Weddell seal (*Leptonychotes weddellii*). Communications Biology, 7, Article 1394. 39472475 10.1038/s42003-024-07029-0 PMC11522681

[cobi70098-bib-0024] Smith, C. R. , Rowles, T. K. , Gomez, F. M. , Ivančić, M. , Colegrove, K. M. , Takeshita, R. , Townsend, F. I. , Zolman, E. S. , Morey, J. S. , Cendejas, V. , Meegan, J. M. , Musser, W. , Speakman, T. R. , Barratclough, A. , Wells, R. S. , & Schwacke, L. H. (2022). Poor pulmonary health in Barataria Bay dolphins in the eight years following the Deepwater Horizon oil spill. Frontiers in Marine Science, 9, Article 975006.

[cobi70098-bib-0025] Soruco Ruiz, J. M. (2024). Denuncian la muerte de un menor de la Chiquitanía a causa de intoxicación por humo . https://www.vision360.bo/noticias/2024/09/26/12549‐denuncian‐la‐muerte‐de‐un‐menor‐de‐la‐chiquitania‐a‐causa‐de‐intoxicacion‐por‐humo

[cobi70098-bib-0026] Urbanski, S. P. , Hao, W. M. , & Baker, S. (2008). Chemical composition of wildland fire emissions. In A. Bytnerowicz , M. J. Arbaugh , A. R. Riebau , & C. Andersen (Eds.), Developments in environmental science (Vol. 8, pp. 79–107). Elsevier.

[cobi70098-bib-0027] Venn‐Watson, S. , Smith, C. R. , Jensen, E. D. , & Rowles, T. (2013). Assessing the potential health impacts of the 2003 and 2007 firestorms on bottlenose dolphins (*Tursiops trucatus*) in San Diego Bay. Inhalation Toxicology, 25, 481–491. 23905966 10.3109/08958378.2013.804611

[cobi70098-bib-0028] Wells, R. S. , & Fahlman, A. (2024). Human impacts on dolphins: Physiological effects and conservation. In A. Fahlman & S. K. Hooker (Eds.), The physiology of dolphins (pp. 267–281). Academic Press.

[cobi70098-bib-0029] Zhang, Y. , Xu, R. , Huang, W. , Ye, T. , Yu, P. , Yu, W. , Wu, Y. , Liu, Y. , Yang, Z. , Wen, B. , Ju, K. , Song, J. , Abramson, M. J. , Johnson, A. , Capon, A. , Jalaludin, B. , Green, D. , Lavigne, E. , Johnston, F. H. , … Li, S. (2025). Respiratory risks from wildfire‐specific PM₂.₅ across multiple countries and territories. Nature Sustainability, 8, 474–484.

